# SALIVA BASED BIOMARKERS TO IDENTIFY COGNITIVE IMPAIRMENT IN OLDER ADULTS

**DOI:** 10.1093/geroni/igac059.2930

**Published:** 2022-12-20

**Authors:** Vinod Yata, Shalini Jain, Michal Masternak, Peter Holland, Marc Agronin, Hariom Yadav, MiaGB Consortium Team

**Affiliations:** Center for Excellence of Aging and Brain Repair, USF Health, Tampa, Florida, United States; University of South Florida, Tampa, Florida, United States; University of Central Florida, Orlando, Florida, United States; Florida Atlantic University, Boca Raton, Florida, United States; Miami Jewish Health, Miami, Florida, United States; University of South Florida, Tampa, Florida, United States; University of South Florida, Tampa, Florida, United States

## Abstract

The prevalence of cognitive decline and dementia is increasing in older adults, their prognosis is poor. Multiple emerging evidence shows that early intervention can delay and /or prevent their progression, however, early-detection markers are invasive, expensive, and not easy to routinely measure. Saliva can be an attractive source of early cognitive decline markers because oral health is linked with cognitive function and oral fluids are connected with brain fluids, and abnormal brain protein markers can leak out to saliva and vice-versa. Using global unbiased LC-MS/MS-based proteomics of 22 saliva samples of cognitively impaired and 39 cognitively healthy older adults (>60 years old) from large, multi-site study called Microbiome in aging Gut and Brain (MiaGB) consortium, revealed that 22 proteins were uniquely for cognitively impaired group while 44 were unique for cognitively healthy controls. In addition, among 78 differentially abundant proteins between cognitively impaired and control groups, half (39) were upregulated, and half (39) were downregulated. Notably, unique proteins in saliva of participants with cognitive impairment were from neurological pathways like NGF signaling, mTOR signaling and LPS-stimulated MAPK signaling. In addition, differentially abundant proteins in participants with cognitive impairment enriched with glucocorticoid receptor signaling, LXR/RXR activation, and L-DOPA degradation-pathways, while they were deficient of pathways like complement C3 and lysozyme pathways. We discovered that the novel saliva proteins and their pathways could blindly differentiate cognitive impaired from healthy older adults. These data suggest that we discovered novel saliva-based proteins that can be used as biomarker to predict/diagnose cognitive-impairment in older adults.

